# Supplementary material for: Metabolic Defects Caused by High-Fat Diet Modify Disease Risk through Inflammatory and Amyloidogenic Pathways in a Mouse Model of Alzheimer’s Disease
Source: Nutrients. 2020 Sep 29;12(10):2977. doi: 10.3390/nu12102977 (PMC7600118; doi:10.3390/nu12102977)
Supplement: Supplementary file 1 [file nutrients-12-02977-s001.pdf]

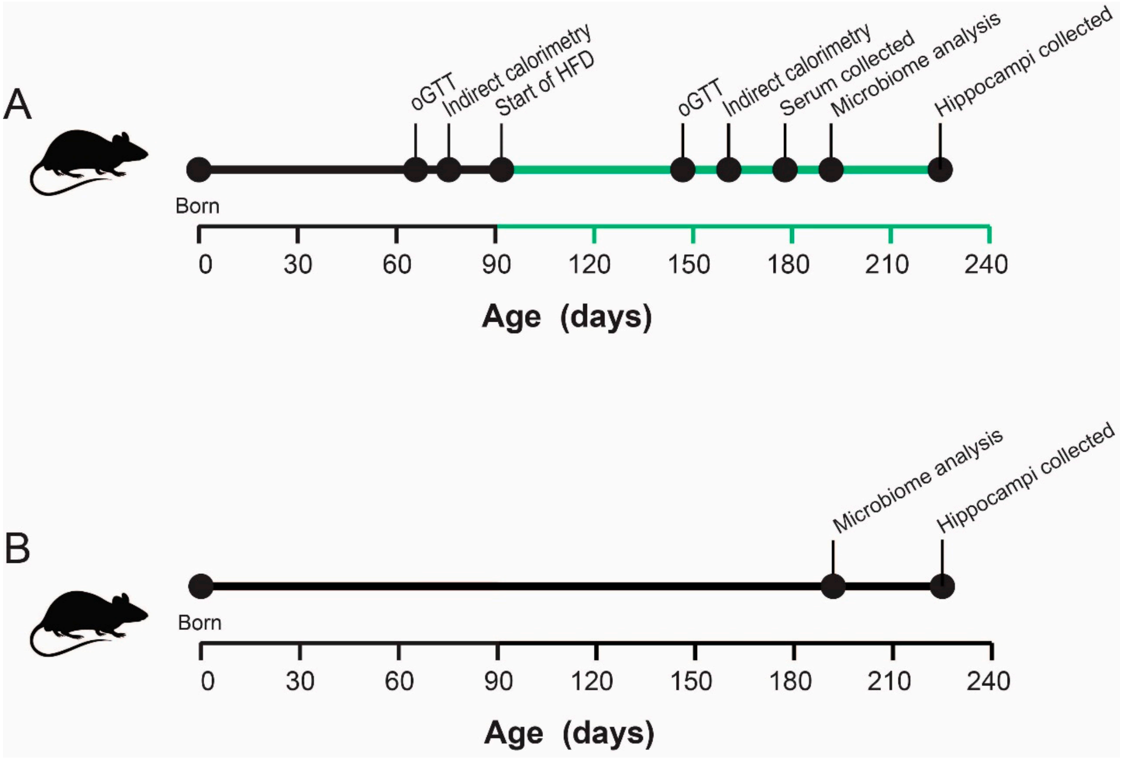

Supplemental Figure S1: (A) Metabolic assays were performed before and after HFD exposure in age-matched 5xFAD and WT mice. Serum, feces, and hippocampi were collected at the timepoints indicated. (**B**) A separate cohort of age-matched 5xFAD and WT mice were fed NCD only. Feces and hippocampi were collected at ages matching the HFD-fed cohort in panel A.

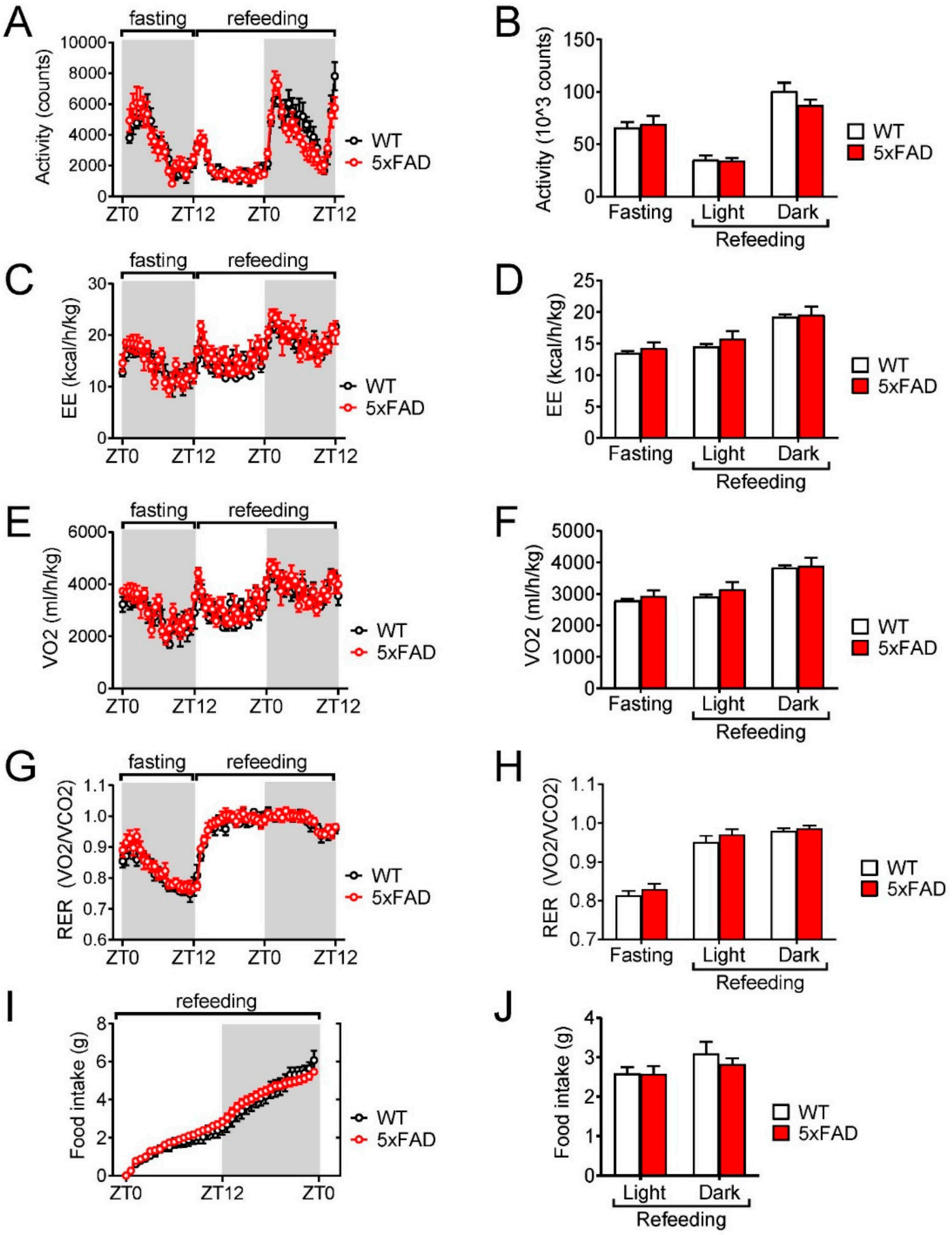

Supplemental Figure S2: Indirect calorimetry during fasting/refeeding challenge in male 5xFAD and WT mice fed NCD. Male 5xFAD and WT mice were 2 months old and fed normal chow diet (NCD). Metabolic profiling was performed during fasting and refeeding. (**A**) Lateral locomotor activity and (**B**) total locomotor activity. (**C**) Energy expenditure (EE) normalized to total body weight and (**D**) average EEEE normalized to total body weight. (**E**) Oxygen consumption (VO2) and (**F**) average VO2. (**G**) Respiratory exchange ratio (RER) and (**H**) average RER. (**I**) Cumulative food intake and (**J**) total food intake during each light/dark phase. Data are displayed as means ± SEM. Statistical comparisons for time-course data were calculated with Fisher's LSD. Statistical comparisons for light/dark averages were calculated with student's t-test. n = 5 for WT mice and n = 6 for 5xFAD mice.

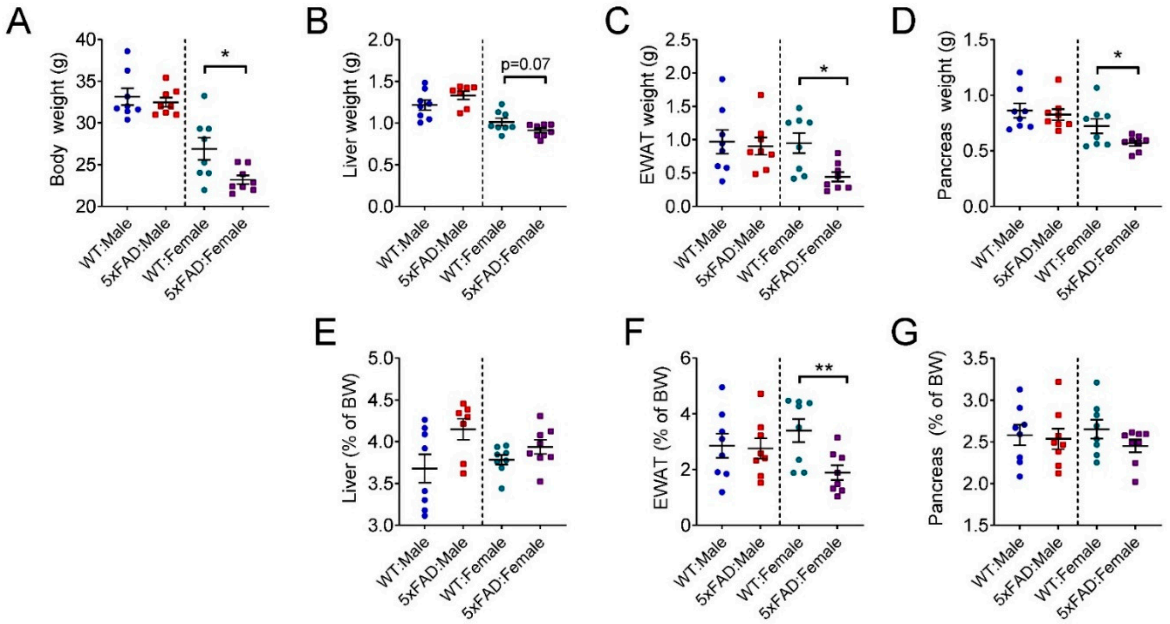

Supplemental Figure S3: 5xFAD mice fed normal chow diet exhibited age-dependent sexually dimorphic effects on body weight maintenance. 5xFAD female mice fed NCD have altered body composition. (**A**) Body weight. (**B**) Liver weight. (**C**) Epididymal white adipose tissue (EWAT) weight. (**D**) Pancreas weight. (**E**) Liver weight as a percentage of body weight (BW). (**F**) Epididymal white adipose tissue (EWAT) weight as a percentage of body weight (BW). (**G**) Pancreas weight as a percentage of body weight (BW). Statistical comparisons between genotypes of same sex were calculated with student's t-test. (*) indicates $p < 0.05$; (**) indicates $p < 0.01$. A, C, D, F, G: $n = 8, 8, 8, 8$; panel B&E: $n = 8, 7, 8, 8$.

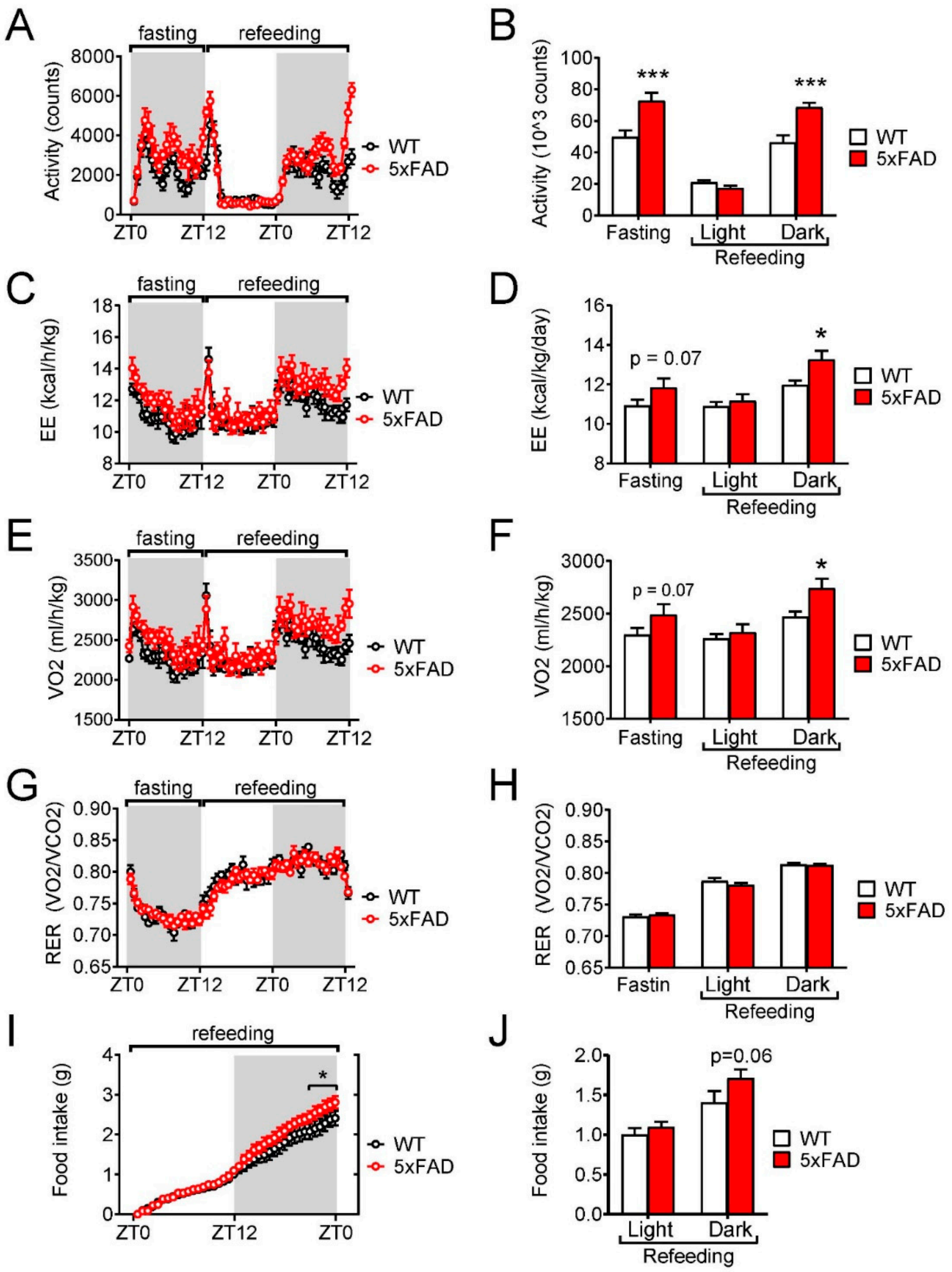

Supplemental Figure S4: Indirect calorimetry during fasting/refeeding challenge in male 5xFAD and WT mice fed high-fat diet (HFD). Male 5xFAD mice were fed HFD for 2 months. Metabolic profiling was performed during fasting/refeeding on HFD. (**A**) Lateral locomotor activity and (**B**) total locomotor activity. (**C**) Energy expenditure (EE) normalized to total body weight and (**D**) average EE normalized to total body weight. (**E**) Oxygen consumption (VO2) and (**F**) average VO2. (**G**) Respiratory exchange ratio (RER) and (**H**) average RER. (**I**) Cumulative food intake and (**J**) total food intake during each light/dark phase. Data are displayed as means ±SEM. Statistical comparisons for time-course data were calculated with Fisher's LSD. Statistical comparisons for light/dark averages were calculated with student's t-test. Statistical comparisons for bar graphs were calculated with student's t-test. (*) indicates $p < 0.05$; (***) indicates $p < 0.001$. $n = 5$ for each group.

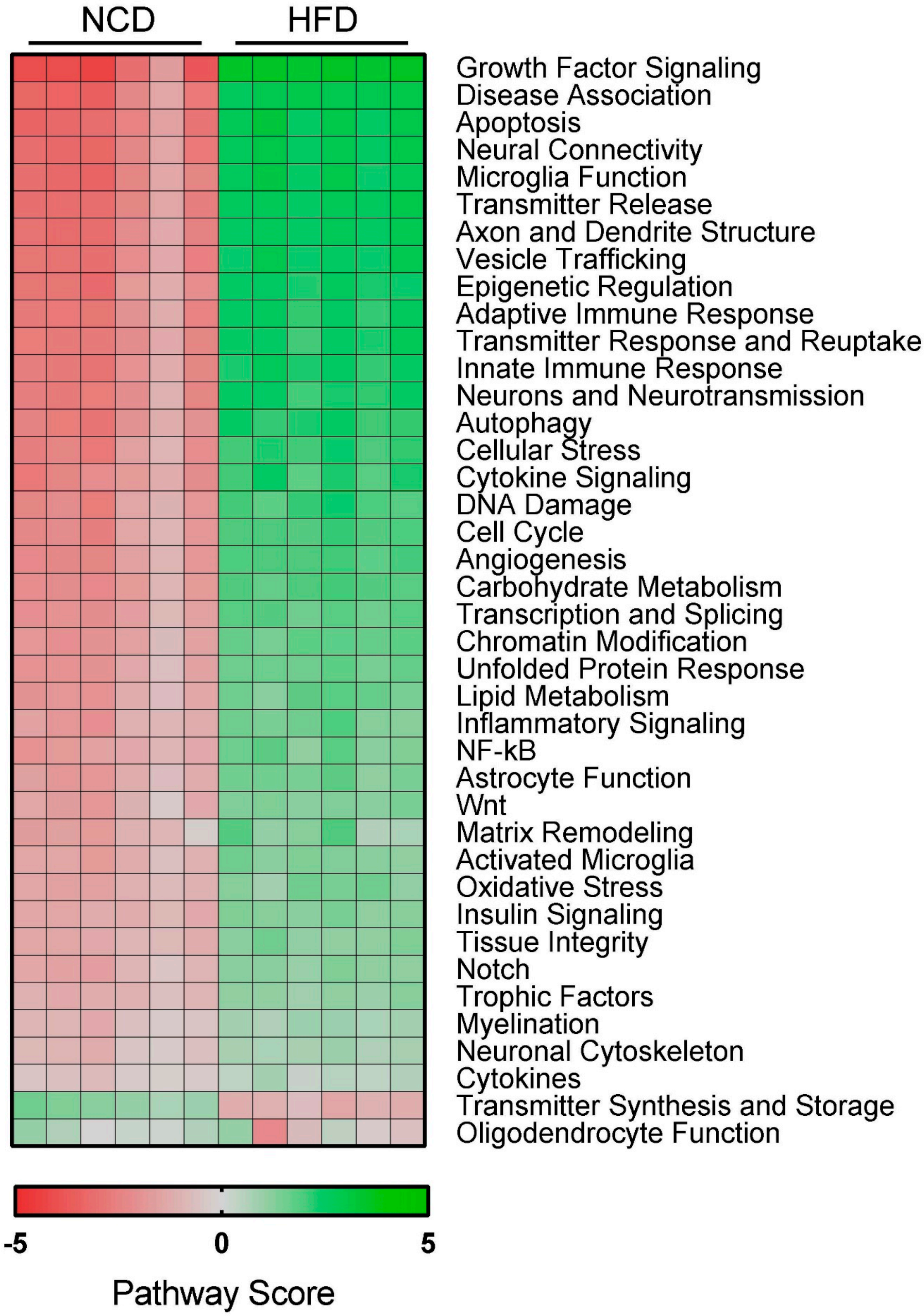

Supplemental Figure S5: Heatmap of Nanostring pathway scores of 5xFAD mice on NCD versus HFD. RNA profiling was performed using samples isolated from the hippocampi of WT and 5xFAD mice fed NCD and HFD. Each column corresponds to a single biological sample. Pathways are ranked by root mean square. n = 6 for each group.
